# Hijacking Complement Regulatory Proteins for Bacterial Immune Evasion

**DOI:** 10.3389/fmicb.2016.02004

**Published:** 2016-12-20

**Authors:** Elise S. Hovingh, Bryan van den Broek, Ilse Jongerius

**Affiliations:** ^1^Department of Medical Microbiology, University Medical Center UtrechtUtrecht, Netherlands; ^2^Centre for Infectious Disease Control, National Institute for Public Health and the EnvironmentBilthoven, Netherlands

**Keywords:** complement regulatory proteins, immune evasion of bacteria, bacteria, vaccine development, complement

## Abstract

The human complement system plays an important role in the defense against invading pathogens, inflammation and homeostasis. Invading microbes, such as bacteria, directly activate the complement system resulting in the formation of chemoattractants and in effective labeling of the bacteria for phagocytosis. In addition, formation of the membrane attack complex is responsible for direct killing of Gram-negative bacteria. In turn, bacteria have evolved several ways to evade complement activation on their surface in order to be able to colonize and invade the human host. One important mechanism of bacterial escape is attraction of complement regulatory proteins to the microbial surface. These molecules are present in the human body for tight regulation of the complement system to prevent damage to host self-surfaces. Therefore, recruitment of complement regulatory proteins to the bacterial surface results in decreased complement activation on the microbial surface which favors bacterial survival. This review will discuss recent advances in understanding the binding of complement regulatory proteins to the bacterial surface at the molecular level. This includes, new insights that have become available concerning specific conserved motives on complement regulatory proteins that are favorable for microbial binding. Finally, complement evasion molecules are of high importance for vaccine development due to their dominant role in bacterial survival, high immunogenicity and homology as well as their presence on the bacterial surface. Here, the use of complement evasion molecules for vaccine development will be discussed.

## Introduction

## The complement system

The complement system is a complex innate immune surveillance system responsible for the defense against invading microbes, inflammation and homeostasis of the host (Merle et al., 2015). In addition, innate pattern recognition receptors, including the Toll-like receptors (TLRs) are necessary to sense invading microbes. During an infection, the complement system is of great importance to label bacteria for phagocytosis, kill them directly via pore formation and to stimulate B-cells via C3d, bridging the innate and adaptive immune system. Moreover, cross-talk between the complement system and TLRs is essential to obtain an optimal response against the invading microbes. Finally, intracellular complement activation in T-cells is important for the regulation of the adaptive immune response (reviewed Freeley et al., 2016; Hajishengallis and Lambris, 2016). The complement system is the first line of defense against invading microbes. Activation of the complement system occurs via three different pathways namely, the classical pathway (CP), the lectin pathway (LP) and the alternative pathway (AP) and results in opsonization of microbes for phagocytosis, formation of chemoattractants and lysis of Gram-negative bacteria. The CP is activated when IgG or IgM bound to the bacterial surface is recognized by C1q. C1q binding to antibodies results in activation of the two associated serine proteases C1r and C1s (Gaboriaud et al., 2004). Activated C1s cleaves C4 into the anaphylatoxin C4a and C4b, of which the latter will covalently attach to the bacterial surface via its exposed thioester domain (Law and Dodds, 1997). Subsequently, C2 binds to C4b and is cleaved by C1s into C2a and C2b resulting in the formation of the CP C3 convertase C4b2a (Merle et al., 2015). Activation of the LP results in the formation of the same C3 convertase (C4b2a) as the CP, however the initiation of the LP differs from the CP. The LP is activated when Mannan-binding lectin (MBL), ficolins and/or collectin 11 recognize specific sugar patterns on the microbial surface (Holmskov et al., 2003; Hansen et al., 2010). Upon binding of lectins, the MBL-associated serine proteases (MASPs) are activated. MASP-2 is solely responsible for cleavage of C4 while C2 is cleaved by MASP-2 and MASP-1 (Drentin et al., 2015). The C3 convertase of the CP/LP (C4b2a) cleaves C3 into the anaphylatoxin C3a and C3b which will covalently deposit on the bacterial membrane via its exposed thioester domain (Law and Dodds, 1997). The AP can be activated spontaneously via hydrolysis of C3. C3(H_2_0) can form the basis for the start of the AP. However, the AP is mainly activated upon binding of deposited C3b therefore, the AP mainly functions as an amplification loop of the CP and the LP (Merle et al., 2015). Factor B (FB) binds to the covalently bound C3b on the microbial surface and is cleaved by factor D (FD) into Bb and Ba, resulting in formation of the AP C3 convertase C3bBb (Forneris et al., 2010). The AP is responsible for a very important proportion of the total complement activation (Harboe et al., 2004; Harboe and Mollnes, 2008). Properdin is the only known positive regulator of the complement system extending the half-life of the AP C3 convertases. In addition, several observations indicate that properdin can also act as a selective pattern recognition molecule serving as a platform for activation of the AP (Spitzer et al., 2007; Cortes et al., 2012).

All complement activation pathways converge at the formation of the C3 convertases which cleave C3 into C3a and C3b. Opsonization of the bacterial surface with C3b is important for phagocytosis of the pathogen (Merle et al., 2015). In addition, C3b incorporation into C3 convertases results in the formation of C5 convertases. Like the C3 convertases, two different C5 convertases exist namely, the CP/LP C4b2aC3b and the AP C3bBbC3b (Merle et al., 2015). C5 convertases cleave C5 into the strong anaphylatoxin C5a and C5b. C5b forms a soluble complex with C6 which in turn can bind C7. The C5b67 complex has a more lipophilic character and can bind to the cell membrane. After C5b67 is bound to the membrane, C8 binding to this complex leads to the penetration of the lipid bilayer and together with multiple copies of C9 a tubular pore, called the membrane attack complex (MAC), is formed that can lyse Gram-negative bacteria (Bubeck, 2014).

## Host proteins for complement regulation

**Figure 1:**
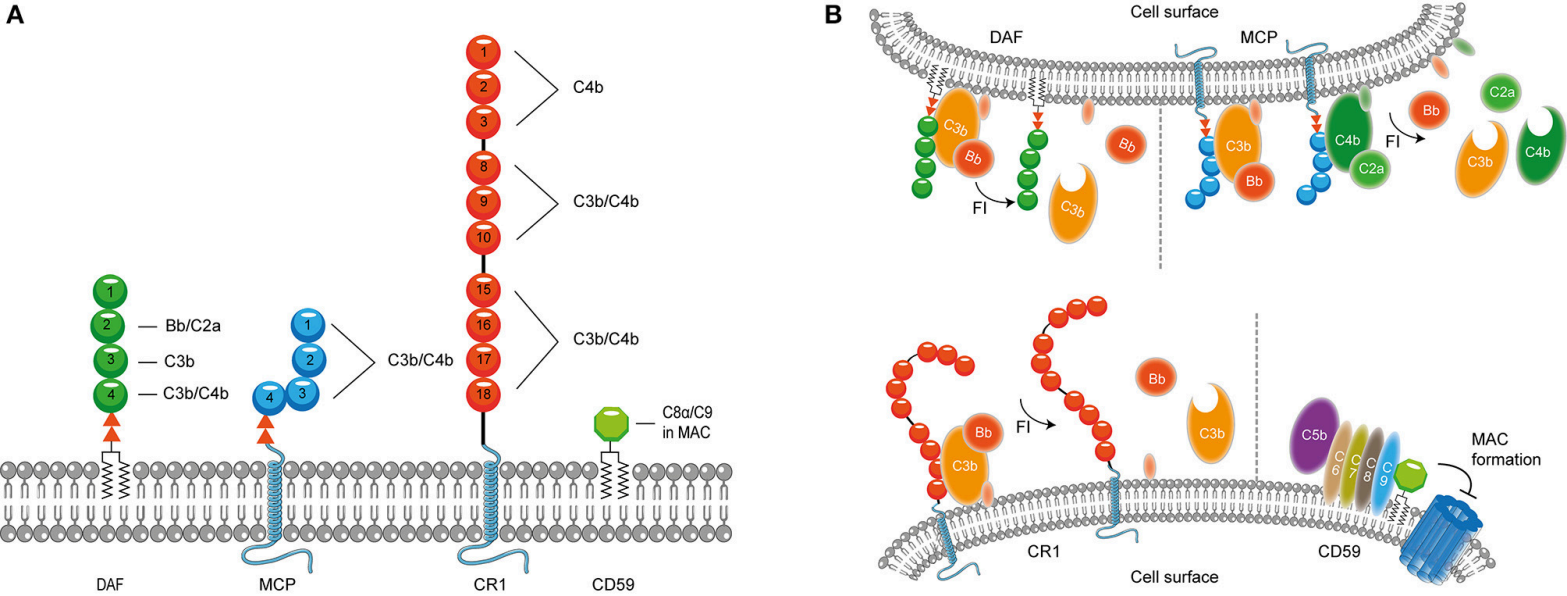
**Membrane bound complement regulatory proteins. (A)** Membrane bound complement regulatory receptors DAF, MCP, CR1, and CD59. The SCRs important for the binding of complement targets are indicated. **(B)** DAF, depicted in the upper membrane, accelerates the decay of C3 convertases and prevents formation of new C3 convertases. MCP, depicted next to DAF, acts as a cofactor for FI, which cleaves C3b and C4b. On the lower membrane, CR1 is shown which acts as a cofactor for FI mediating cleavage of C3b as well as C4b (not depicted in this picture). Lastly, CD59 binds to C5b-8 and C5b-9 preventing the formation of the MAC. Figure was produced using Servier Medical Art.

**Figure 2:**
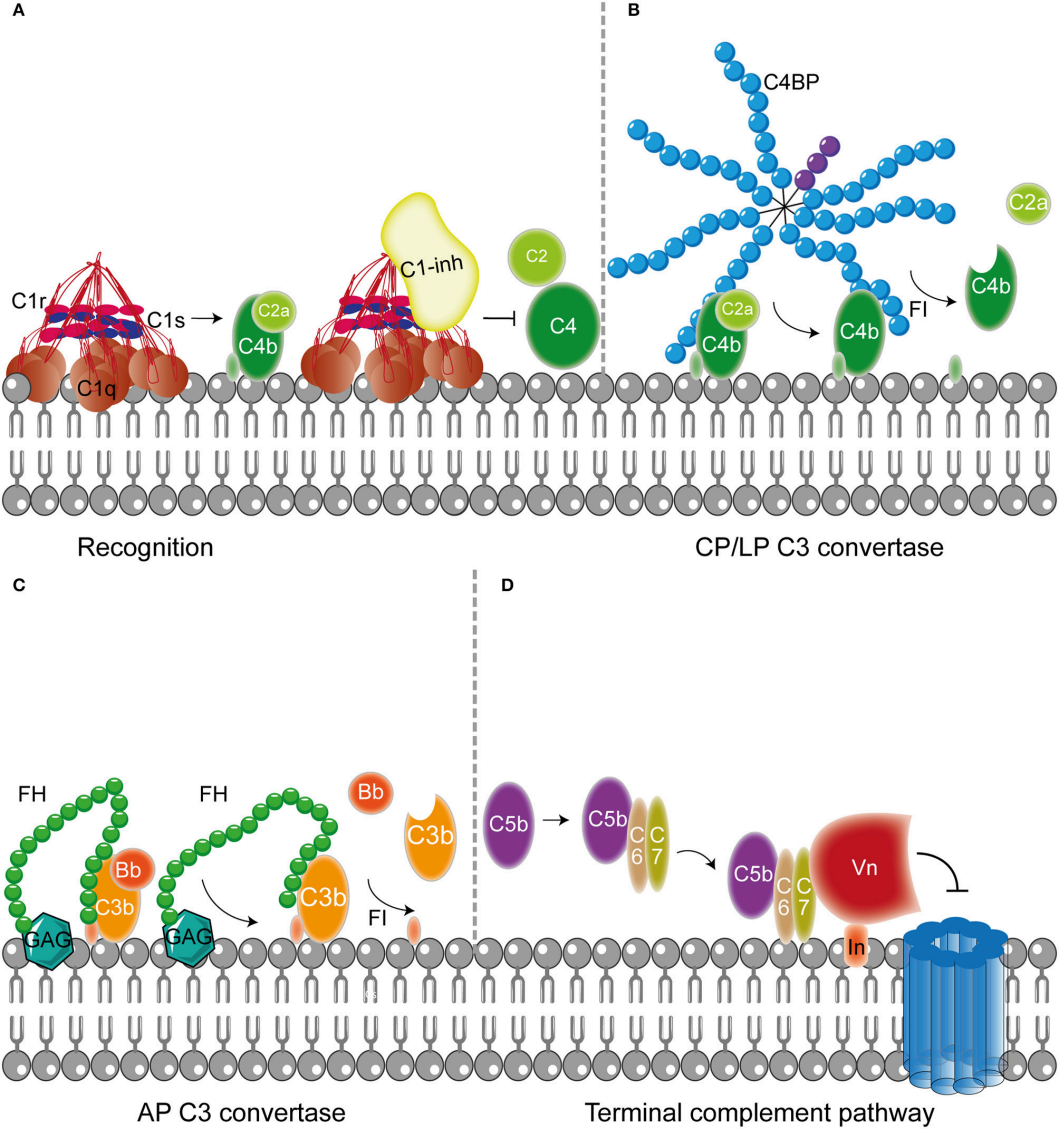
**Host fluid phase complement regulatory proteins. (A)** Fluid regulatory protein C1-inh prevents complement activation by binding to C1s and C1r (and MASP-1 and MASP-2, not depicted here) preventing the cleavage of C4 and C2 and hence the formation of the CP and LP C3 convertase. **(B)** C4BP binding to C4b mediates dissociation of the CP and LP C3 and C5 convertases. Furthermore, C4BP binding to C4b increases proteolytic cleavage of C4b by FI. **(C)** The main AP fluid regulator, FH, binds to GAG on the cellular membrane and mediates dissociation of the AP C3 convertases by binding C3b causing dissociation of Bb from the C3 convertase. Furthermore, FI is recruited leading to the proteolytic cleavage of C3b. **(D)** The last fluid phase inhibitor is Vn which binds to integrins in the host membrane. Vn binds the metastable membrane binding site of C5b-7 preventing the formation of the MAC. Figure was produced using Servier Medical Art.

It is important to confine complement activation to pathogenic surfaces due to the destructive potential of complement activation to the host. Therefore, effectors of complement activation need to be tightly regulated by complement regulatory proteins to maintain the balance between efficient destruction of pathogens and prevention of unwanted activation on the host tissue (Merle et al., 2015). Complement regulatory proteins are either soluble, and present in plasma, or membrane bound. Most, but not all, complement regulatory proteins consist of individually folded short consensus repeats (SCRs) (Figures 1, 2) containing approximately 60 amino acids (aa) per SCR including four conserved cysteine residues that form intradomain bonds (Holers et al., 1985; Merle et al., 2015). Four membrane bound regulatory proteins are known namely, decay accelerating factor (DAF or CD55), membrane cofactor protein (MCP or CD46), complement receptor 1 (CR1 or CD35) and CD59 (Merle et al., 2015) (Figure 1A).

DAF is a glycosylphosphatidyl-inositol (GPI) anchored complement regulatory protein that consists of four extracellular SCRs. A Ser/Thr-rich region is present between the four SCRs and the GPI anchor. DAF is widely expressed on hematopoietic and non-hematopoietic cells. This protein decreases complement activation on the surface by accelerating the decay of C3 convertases. Moreover, DAF also prevents the assembly of C3 convertases (Figure 1B) (Lublin and Atkinson, 1989). While DAF mediates the decay of the CP/LP C3 convertase using SCRs 2-3 (Brodbeck et al., 1996; Kuttner-Kondo et al., 2003) the protein needs SCRs 2-4 for decay of the AP. Studies have shown that the binding of SCR 4 to C3b directly mediates decay acceleration of the AP C3 convertase and that SCR 2 binding to Bb increases avidity of DAF binding to the convertase (Harris et al., 2005, 2007). The second membrane bound complement regulator described here is the ubiquitously expressed MCP. MCP is linked to the membrane through transmembrane domains. Like DAF, the regulator consist of four extracellular SCR domains and contains a Ser/Thr rich region between the SCRs and the membrane anchor (Kim and Song, 2006). MCP serves as a cofactor for factor I (FI) (which will be discussed in more detail below) mediated cleavage of C3b and C4b (Barilla-LaBarca et al., 2002). Studies indicate that SCRs 2-4 of MCP are important for the binding to C3b and C4b. However, the most important binding site for C3b is located on SCR 4 (Figure 1A; Persson et al., 2010).

CR1 consists of 30 SCRs and is bound to the cell surface via a transmembrane domain and has a cytoplasmic tail. CR1 is expressed on leukocytes, erythrocytes and glomerular podocytes. The first 28 SCRs of CR1 consist of four long and highly homologous repeats of seven SCRs (Wong et al., 1989). Similar to MCP, CR1 is a cofactor for FI-mediated cleavage of C4b and C3b (Figure 1). C4b and C3b bind to SCRs 8-10 and SCRs 15-17 where the latter site has a major role in C3b/C4b inactivation (Smith et al., 2002). In addition, SCRs 1-4 bind C4b only (Reilly et al., 1994). The final membrane bound regulator described here is CD59. CD59 is a glycoprotein that consist of 77 aa and is bound to the membrane via a GPI anchor (Wickham et al., 2011). Structurally, CD59 has no similarity with other complement proteins. CD59 is expressed on a wide range of cells, where it binds to C5b-8 and C5b-9 preventing the formation of the MAC in the lipid bilayer. CD59 cannot bind to free C8 or C9, but specifically interacts with the MAC/perforin (MACPF) domain of each protein that becomes available upon complex formation (Figure 1B; Farkas et al., 2002; Wickham et al., 2011).

In addition to membrane bound complement regulators, several soluble complement regulators have been described (for details regarding their weight and plasma concentration see Table 1). In contrast to the membrane bound regulators, the soluble complement regulators have a broader range of action. C1 inhibitor (C1-inh) is a serine protease inhibitor (serpin) which is involved in protease inhibition. The C-terminal domain of C1-inh is the serine protease inhibitor domain, which is similar to other serpin proteins. This domain contains the protease recognition center. The N-terminal domain is heavily glycosylated, does not share homology with other serpins and plays no role in the protease inhibitory function of C1-inh (Davis et al., 2010). C1-inh inhibits C1r, C1s, MASP1 and MASP-2 thereby preventing activation via the CP (Figure 2A) and the LP. Protease inhibition by C1-inh involves two steps. First, the protease binds to the protease recognition center of the serpin which mimics the substrate. Upon this binding, the protease cleaves the Arg444-Thr445 peptide bond in the protease recognition center. Second, this cleavage triggers a conformational change after which a covalent bond between the serpin and the protease is formed (Huntington et al., 2000). Another soluble complement inhibitor that inhibits the CP and the LP is C4b binding protein (C4BP). The major isoform is made up of seven identical α-chains and one β-chain. The α-chain consists of eight SCRs and a C-terminal oligomerization domain of approximately 60 aa, while the β-chain consists of three SCRs and a C-terminal oligomerization domain (Dahlbäck et al., 1983). The assembly results in the characteristic spider-like structure of the C4BP complex (Hofmeyer et al., 2012). C4BP binds C4b via positively charged residues in SCR 1-3 of the α-chain. Although all 7 α-chains are capable of C4b binding only four C4b molecules can bind at once due to steric hindrance (Ziccardi et al., 1984; Blom et al., 1999). Binding of C4BP to C4b results in increased proteolytic cleavage of C4b by FI and also inhibits the assembly of the C3 and C5 convertases of the CP and the LP (Figure 2B; Rawal et al., 2009). Next to binding to C4b, C4BP can also bind to C3b and C2a albeit to a lesser extent (Hofmeyer et al., 2012).

**Table 1:** **Overview of molecular mass and plasma concentrations of soluble complement regulatory proteins**.

**Complement regulator**	**Molecular mass**	**Plasma concentration**	**References**
C1-inh	105 kDa	250 μg/ml	Davis et al., 2010
C4BP	570 kDa	200 μg/ml	Dahlbäck et al., 1983
FH	150 kDa	400–500 μg/ml	Friese et al., 2000
FHL-1	42 kDa	30–50 μg/ml	Friese et al., 2000
Clusterin	80 kDa	50–200 μg/ml	Rull et al., 2015
Vn	70 kDa	± 500 μg/ml	Boyd et al., 1993

Factor H (FH) and Factor H like protein 1 (FHL-1) are the main fluid phase regulators of the AP. The 150 kD glycoprotein FH, consists of 20 SCRs. FHL-1 is a 43 kDa product of an alternatively spliced transcript of the FH gene which is made up of the seven N-terminal SCRs domains of FH with a unique C-terminal extension of four hydrophobic aa residues. As fluid phase regulators of the AP, FH and FHL-1 prevent the formation of C3 convertases, accelerate the decay of the AP C3 convertase and act as cofactors for protease FI, which results in the cleavage of C3b to the inactive iC3b (Wu et al., 2009; Ferreira et al., 2010). The regulatory activity of FH is mediated by SCRs 1-4 (Gordon et al., 1995) (Figure 2C). FI is an 66 kDa serine protease that regulates the formation of C3 convertases (Tsiftsoglou et al., 2006). FI specifically cleaves two arginyl peptide bonds in the α or α' chain of C3b and C4b, thereby preventing formation of C3 and C5 convertases (Fujita et al., 1978; Davis and Harrison, 1982). This proteolysis can only occur in the presence of co-factors described above namely FH, C4BP, MCP, and CR1. FI circulates in plasma in a proteolytically inactive form despite being processed to the mature sequence. Only after binding to the substrate and a co-factor, FI becomes active (Roversi et al., 2011).

Besides regulation of the convertases, formation of the MAC is under tight control as well. The MAC is regulated by two soluble complement regulatory proteins namely clusterin and vitronectin (Vn). Clusterin consists of an α- and a β-chain linked via disulphide bounds (Wilson and Easterbrook-Smith, 2000). Clusterin binds to C7 and can prevent membrane attachement, in addition it can bind to C8β and C9 to prevent C9 assembly (McDonald and Nelsestuen, 1997). Vn is a multifunctional glycoprotein that inhibits the formation of the MAC (Morgan, 1999) by preventing the insertion of the MAC by associating with the metastable membrane binding site of C5b-7 (Preissner, 1991; Figure 2D). In the presence of Vn, soluble C5b-7 remains capable of binding to C8 and C9 however the subsequently formed complex is incapable of lysing bacteria. The heparin binding domain of Vn was shown to bind C5b-9, thereby preventing C9 polymerization (Milis et al., 1993).

## Bacterial complement evasion strategies

**Figure 3:**
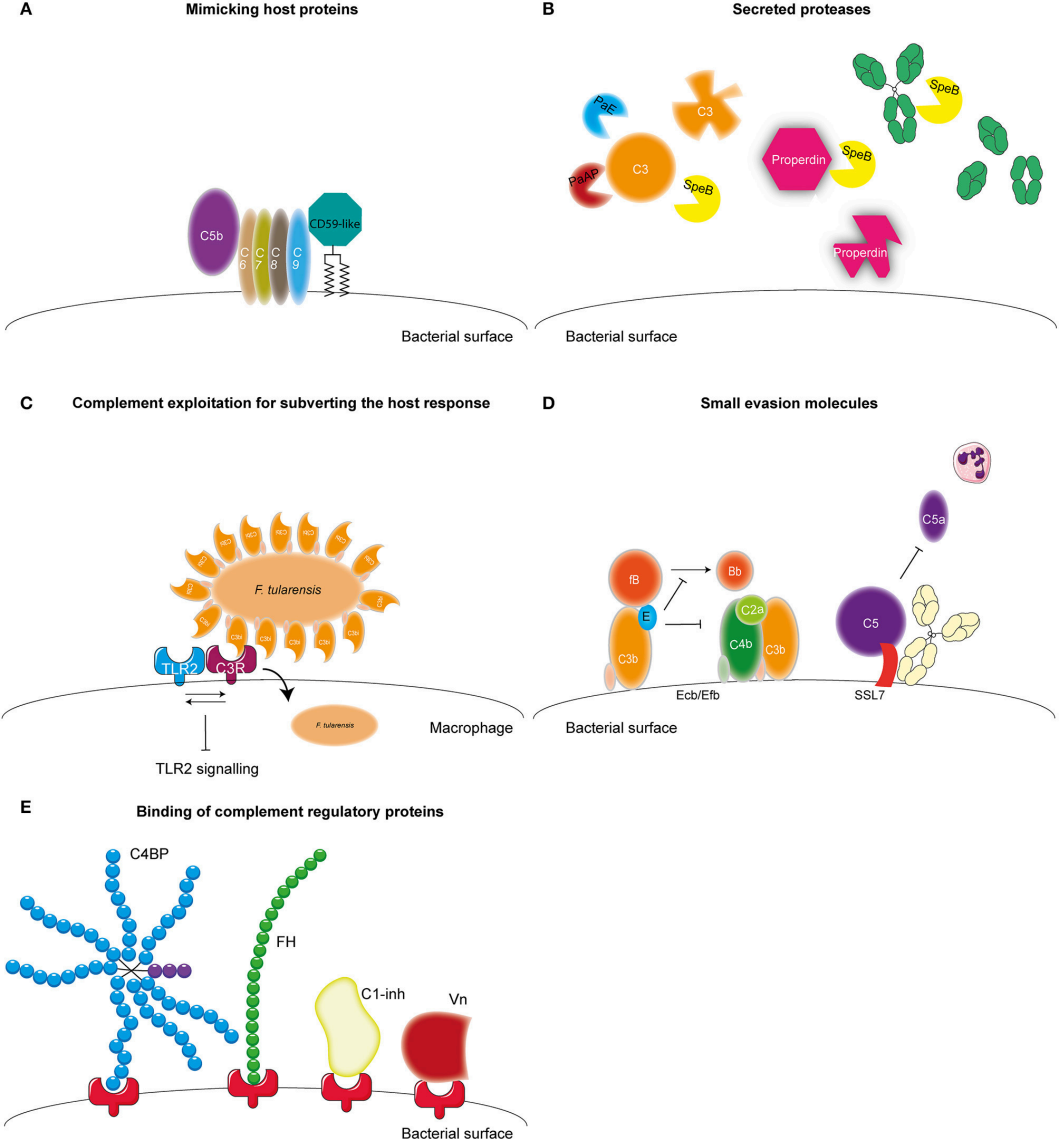
**Complement evasion strategies of bacteria**. Complement evasion strategies of bacteria can be divided into four different groups. **(A)** The expression of proteins that mimic host structures like the CD59-like protein of *B. burgdorferi* which inhibits MAC formation. **(B)** The secretion of bacterial proteases like PaE and PaAP expressed by *P. aeruginosa* which cleave C3. *S. pyogenes* SpeB cleaves C3, properdin and IgG. **(C)** Inhibition of TLR2 by *F. tularensis*. This bacterium exploits being opsonized by C3b to engage CR3 on macrophages to facilitate entry as well as to induce both inside-out as well as outside-in signaling of CR3 resulting in decreased TLR2 activity. **(D)** The secretion of small bacterial proteins with complement inhibitory properties as is shown for *S. aureus*. This pathogen secretes Ecb and Efb that bind to the C3d domain of C3b and thereby inhibit the formation of the C3b containing CP and LP C5 convertase as well as inhibit the formation of the AP C3 convertase by stabilizing the binding of FB to C3b preventing cleavage of FB into Bb. Furthermore, SSL7 is secreted which binds C5 and IgA inhibiting the generation of C5a and thus subsequent neutrophil infiltration. **(E)** The recruitment of fluid phase complement regulatory proteins C1-inh, C4BP, FH, and Vn to the bacterial membrane which will be discussed in this review. Figure was produced using Servier Medical Art.

Microbes have developed several mechanisms to interfere with the complement system in order to survive in the host. Most bacteria use multiple of these mechanisms to obstruct this system (Lambris et al., 2008). The first mechanism of complement evasion described here is mimicking of complement regulatory proteins (Figure 3A). The only bacterium known to use this mechanism is *Borrelia Burgdorferi*. *B. Burgdorferi* is a Gram-negative bacterium and the causative agent of Lyme disease, which is one of the major emerging arthropod borne infections in the world (Hengge et al., 2003). *B. burgdorferi* expresses the 80 kDa “CD59-like” protein on its membrane that binds to C8b and C9 in complex and inhibits MAC formation (Pausa et al., 2003). With a molecular weight of 80 kDa this protein is much larger than the human CD59, however there is some structural similarity since polyclonal anti-CD59 antibody was able to recognize CD59-like (Pausa et al., 2003). Although, CD59-like of *B. burgdorferi* is the only example of complement regulator mimicry by bacteria, this mechanism is widely used among viruses (Liszewski et al., 2006; Bernet et al., 2011).

A second mechanism that is used by bacteria to evade the complement system is the expression of proteases which can cleave complement components (Figure 3B). *Pseudomonas aeruginosa* is a Gram-negative opportunistic pathogen. It mainly causes infections in patients suffering from cystic fibrosis, chronic obstructive pulmonary disease as well as ventilator associated pneumonia and is the main causative agent of hospital acquired pneumonia and septicemia (Döring et al., 2011). *P. aeruginosa* secretes two proteases that cleave complement components namely, Pseudomonas elastase (PaE) and Pseudomonas alkaline protease (PaAP). Both proteins degrade C1q and C3, thereby preventing complement activation on the bacterial surface (Rooijakkers and van Strijp, 2007; Merle et al., 2015). Another pathogen that is known to cleave complement components using proteases is *Streptococcus pyogenes*. *S. pyogenes* is a Gram-positive bacteria also known as group A Streptococcus (GAS). GAS is responsible for a wide spectrum of disease ranging from strep throat to life-threatening necrotizing fasciitis (Brouwer et al., 2016). GAS expresses Streptococcal pyrogenic exotoxin B (SpeB), an endoprotease that can cleave a wide range of proteins including C3, immunoglobulin (Ig) and properdin (von Pawel-Rammingen and Björck, 2003; Tsao et al., 2006; Honda-Ogawa et al., 2013). SpeB cleaves IgG at the hinge region resulting in two Fab and one Fc region preventing IgG-mediated phagocytosis (von Pawel-Rammingen and Björck, 2003). Cleavage of properdin, the positive regulator of complement activation, and C3 by SpeB interferes with the formation of C3 convertases and reduces opsonization of bacteria (Honda-Ogawa et al., 2013). The expression of proteases that cleave complement proteins is not limited to *P. aeruginosa* and GAS but a widely used mechanism (reviewed in Potempa and Potempa, 2012).

Another pathogen that expresses proteases capable of cleaving complement proteins is *Porphyromonas gingivalis*. This Gram-negative anaerobic bacterium causes periodontitis but can also be involved in systemic diseases such as arthrosclerosis and aspiration pneumonia (Pihlstrom et al., 2005). Instead of degradation of complement components, gingipains of this bacterium cleave C5 into C5a and C5b much like the host C5 convertase (Wingrove et al., 1992). Recent studies show that this increased generation of C5a is used by this pathogen to stimulate the regulatory cross-talk between the C5a receptor and TLR2 on macrophages resulting in a blunted TLR2 response and thus immune suppression (Wang et al., 2010). Inhibition of TLR2 signaling via manipulation of complement has also been reported for *Francisella tularensis*, a zoonotic pathogen causing the severe disease tularemia. This bacterium exploits being opsonized by C3 to engage complement receptor 3 (CR3) on macrophages to facilitate entry into these cells as well as to induce both inside-out as well as outside-in signaling of this receptor resulting in decreased TLR2 activity (Dai et al., 2013) (Figure 3C). Spores from *Bacillus anthracis*, the Gram-positive causative agent of anthrax, additionally manipulate complement for increased uptake. The Bacillus collagen like protein of *B. anthracis* recruits C1q to the surface of the spore leading to C3bi deposition and subsequent CR3 mediated uptake of the spore by macrophages (Gu et al., 2012).

Besides cleavage of complement components, bacteria can also express small evasion molecules that inhibit the complement system (Figure 3D) (for review see Lambris et al., 2008; Laarman et al., 2010; Okumura and Nizet, 2014). Several of those molecules specifically target the central C3 molecule. *Staphylococcus aureus*, is an opportunistic commensal that colonizes 20% of the human population posing a major health risk considering the increase in antibiotic resistant strains. *S. aureus* is a Gram-positive bacterium responsible for an increasing amounts of hospital and community-acquired infections including superficial skin infection for example abscesses, but also serious invasive infections such as endocarditis and sepsis (Okumura and Nizet, 2014). *S. aureus* is a master of secreting small evasion molecules and expresses Extracellular fibrinogen-binding protein (Efb) and Extracellular complement binding protein (Ecb), which both bind to the C3d domain of C3b, thereby inhibiting C3b containing convertases (Jongerius et al., 2007). Moreover, Ecb can form a tripartite complex with FH and C3b on the surface of *S. aureus* (discussed in more detail below) (Amdahl et al., 2013). Inhibition of convertases by Efb and Ecb, results in decreased C3b opsonization on the bacterial surface as well as decreased C5a generation and neutrophil migration toward the site of infection (Jongerius et al., 2007, 2012). Other small secreted molecules of *S. aureus* that target C3 are Staphylococcal complement inhibitor (SCIN) and Surface immunoglobulin-binding protein (Sbi) (Rooijakkers et al., 2005, 2009; Haupt et al., 2008). Furthermore, *S. aureus* also expresses molecules like staphylococcal superantigen like 7 (SSL7) that bind C5 and prevent C5a generation and subsequently decreases neutrophil migration toward the site of infection (Langley et al., 2005). In addition, this protein also binds to IgA. Recent studies show that SSL7 binds C5 and IgA together and efficient inhibition of C5a generation requires both C5 as well as IgA binding (Laursen et al., 2010). Finally, microbes are able to bind (soluble) complement regulators to their surface to limit complement activation on their membrane (Figure 3E) (for review see Kraiczy and Würzner, 2006; Blom et al., 2009 and Table 2). In this review, we will describe newly discovered bacterial proteins that bind complement regulatory proteins and discuss new insights concerning specific conserved motifs on complement regulatory proteins that are favorable for microbial binding. Finally, the use of complement evasion molecules for vaccine development will be discussed as well.

**Table 2:** **Overview of bacterial interactions with host complement regulatory proteins discussed in this review and reviewed in Kraiczy and Würzner (2006) (#) and Blom et al. (2009) ($)**.

**Bacteria**	**Surface ligand**	**Binding domain of ligand**	**Complement regulatory protein**	**Binding domain of complement regulatory protein**	**References**
*B. pertussis*	FHA	Unknown	C4BP	SCRs 1-2 (R64 and R66)	Reviewed in #
	Vag8	Unknown	C1-inh	Serpin domain	Marr et al., 2011
*B. afzelii*	BaCRASP-1	Unknown	FH, FHL-1	SCRs 5-7 and 1-7	Reviewed in #
	BaCRASP-2	Unknown	FH, FHL-1	SCRs 1-7 and 6-7	Reviewed in #
	BaCRASP-3	Unknown	FH	SCRs 6-7	Reviewed in #
	BaCRASP-4	Unknown	FH	SCRs 19-20	Reviewed in #
	BaCRASP-5	Unknown	FH	SCRs 19-20	Reviewed in #
*B. burgdorferi*	CspA	Middle part different regions	FH, FHL-1	SCRs 5-7 and 20	Reviewed in # and $
	CspZ	aa 96-71 and 202-266	FH, FHL-1	SCRs 5-7 and 19-20	Reviewed in # and $
	ErpA	C-terminus	FH, FHR 1,2, and 5	SCRs 19-20	Reviewed in #
	ErpC	C-terminus (loop region)	FHR 1,2, and 5	SCRs 19-20	Reviewed in #
	ErpP	C-terminus (loop region)	FH, FHR 1,2, and 5	SCsR19- 20	Reviewed in #
	OspE	C-terminus (loop region)	FH, FHL-1	SCRs 19-20	Reviewed in #
	Outer surface protein A	Unknown	C4BP	SCRs 1-3	Pietikäinen et al., 2010; Madar et al., 2015
	p21	Unknown	FH	SCRs 19-20	Reviewed in #
	Variable large protein	Unknown	C4BP	SCRs 1-3	Pietikäinen et al., 2010; Madar et al., 2015
	Variable major protein	Unknown	C4BP	SCRs 1-3	Pietikäinen et al., 2010; Madar et al., 2015
	35 kDa protein	Unknown	FH, FHL-1	Unknown	Reviewed in #
*B. hermsii*	FHBP19	Unknown	FH	Unknown	Reviewed in #
	FHBP28	Unknown	FH	Unknown	Reviewed in #
*B. recurrentis*	CihC	Central part	C4BP	Unknown	Grosskinsky et al., 2010
		Central part	C1-inh	Unknown	Grosskinsky et al., 2010
*E. coli*	OmpA	N-terminal region	C4BP	SCR 3 and 8	Reviewed in # and $
	StcE	Unknown	C1-inh	Unknown	Lathem et al., 2002, 2004
*H. influenzae*	Hsf	aa 429-652	Vn	HBD3	Reviewed in $ Singh et al., 2014
	OmpP5	Loop 1 and 2	FH	Unknown	Langereis et al., 2014; Rosadini et al., 2014
	Protein E	aa 84-108	Vn	HBD3	Hallström et al., 2009; Singh et al., 2013
	Protein F	aa 23-48	Vn	HBD3	Su et al., 2013
	Protein H	Unknown	FH, FHL-1	SRCs 7 and 18-20	Fleury et al., 2014
			Vn	HBD3	Al-Jubair et al., 2015
	Unknown	Unknown	C4BP	SCR 2 and 7	Hallström et al., 2007
*M. catarrhalis*	UspA1	Unknown	C4BP	SCRs 2,5, or 7	Reviewed in # and $
	UspA2	Unknown	C4BP	SCRs 2,5, or 7	Reviewed in #
		Unknown	Vn	Unknown	Reviewed in $
*N. gonorroheae*	LOS	Sialic acid	FH	SCRs 16-20	Reviewed in #
	Por1A	Loop5	FH	SCR 6 and SCR16-20	Reviewed in # and Ngampasutadol et al., 2008
		Loop 1	C4BP	SCR 1	Reviewed in # and $
	Por1B	Loops 5 and 6	C4BP	SCR 1	Reviewed # and $
	Type IV pili	N-terminus	FH	SCRs 1-2	Reviewed in $
*N. meningitidis*	fHbp	N- and C-terminal β-barrel	FH	SCRs 6-7	Schneider et al., 2009
	Msf	aa 39-82	Vn	Connecting region and HBD3	Griffiths et al., 2011
	NspA	Loop 3	FH	SCRs 6-7	Lewis et al., 2010
	OpaA	Unknown	Vn	Unknown	Reviewed in $
	OpC	Unknown	Vn	Connecting region and HBD3	Sa E Cunha et al., 2010
	PorA	Unknown	C4BP	SCRs 2-3	Reviewed in $
	Porin B2	Unknown	FH	Unknown	Lewis et al., 2013
*P. aeruginosa*	Lpd	aa 1-160 and 328-478	Vn	HBD3	Hallström et al., 2015
		Unknown	fH	SCRs 7 and 18-20	Hallström et al., 2012
	Porin D	aa 161-287 and 264-478	Vn	HBD3	Paulsson et al., 2015
*S. aureus*	Bone sialoprotein binding protein	Unknown	C4BP	Unknown	Hair et al., 2013
		Unknown	FH	Unknown	Hair et al., 2013
	SrdE	Unknown	C4BP	Unknown	Hair et al., 2013
		Unknown	FH	Unknown	Hair et al., 2013
*S. agalactiae*	b protein	C-terminus	FH	SCR 13 or 20	Reviewed in #
*S. pneumoniae*	Hic	aa 151-200	Vn	HBD3	Kohler et al., 2014
		aa 38-92	FH	SCRs 8-11 and 12-14	Reviewed in #
	LytA	Unknown	C4BP	Unknown	Ramos-Sevillano et al., 2015
	PspC	aa 1-225	FH	SCRs 8-10	Reviewed in # and Herbert et al., 2015
		N-terminal	Vn	HBD3	Voss et al., 2013
	PspC4.4	Unknown	C4BP	SCRs 1-2 and SCR8	Dieudonné-Vatran et al., 2009
	Surface exposed enolase	Unknown	C4BP	SCRs 1-2 and SCR8	Agarwal et al., 2012
*S. pyogenes*	emm5 protein	A-region and C-repeats	FH, FHL-1	SCR 7	Reviewed in #
	emm6 protein	C-repeats and B-region	FH, FHL-1	Unknown	Reviewed in #
	emm18 protein	Unknown	FH, FHL-1	SCR 7	Reviewed in #
	Fba	N-terminus	FH, FHL-1	SCR 7	Reviewed in #
	M proteins	Hypervariable region	C4BP	SCRs 1-2	Reviewed in # and $
*Y. enterocolitica*	YadA	Unknown	FH	Unknown	Reviewed in #
		Unknown	C4BP	SCRs 1-2	Kirjavainen et al., 2008
	Ail	Unknown	C4BP	SCRs 1-3	Kirjavainen et al., 2008
*Y. pestis*	Ail	Unknown	C4BP	SCR 6 and 8	Ho et al., 2012, 2014
*Y. pseudotuberculosis*	Ail	Unknown	C4BP	SCRs 6-8	Ho et al., 2012

## Targeting complement at the CP and LP: C1-inh recruitment

As described above, C1-inh inhibits C1r, C1s, MASP1, and MASP-2 thereby preventing activation via the CP and the LP (Davis et al., 2010). Therefore, binding of C1-inh to the bacterial membrane will result in decreased complement activation which is favorable for survival. *Escherichia coli* O157:H7 is a Gram-negative bacterium causing diarrhea, haemorrhagic colitis and potentially lethal hemolytic uremic syndrome. This bacteria contains the 92 kb pO157 plasmid encoding for multiple putative virulence factors (Burland et al., 1998). One of these virulence factors is secreted protease of C1 inhibitor from enterohemorrhagic *E. coli* (StcE). StcE is a Zn^2+^ metalloprotease that recruits C1-inh to the membrane (Lathem et al., 2004). In addition, StcE is also capable of cleaving C1-inh while other serpin family members are unaffected (Lathem et al., 2002). It is proposed that StcE re-associates with the membrane where is can bind the NH_2_-terminal domain of C1-inh. Membrane bound C1-inh subsequently inactivates C1s and C1r, blocking the activation of the CP. In addition, StcE cleaves C1-inh at the NH_2_-terminus releasing the serpin/serine protease complex (Lathem et al., 2004).

Another Gram-negative bacterium shown to bind C1-inh to its surface is *Bordetella pertussis*. *B. pertussis* is the causative agent of whooping cough, also known as pertussis. Pertussis, which can be fatal in infants, is characterized by hypoxemia, pulmonary hypertension, leukocytosis and bronchopneumonia (Kilgore et al., 2016). A pertussis vaccine has been available since the 1950s nonetheless despite high vaccine coverage the disease has been re-emerging in the past two decades (Locht, 2016). *B. pertussis* is relatively resistant to complement-mediated killing, however, research on complement evasion strategies by this pathogen is limited (Fernandez and Weiss, 1998). *B. pertussis* is able to bind C1-inh to its bacterial surface using the autotransporter protein Vag8. C1-inh binding to the surface increased survival of *B. pertussis* in human serum. Vag8 appears to recruit C1-inh via its serpin domain and did not require the N-linked glycosylation of C1-inh (Marr et al., 2007, 2011). The exact mechanism of complement inhibition via recruitment of C1-inh needs to be further investigated.

The last pathogen known to bind C1-inh is the spirochete *Borrelia recurrentis*. *B. recurrentis* is a Gram-negative bacterium and the causal agent of louse-borne relapsing fever. This bacterium is transmitted to humans via the infected body louse *Pediculus humanus*. *B. recurrentis* expresses an outer membrane lipoprotein, namely CihC, that can bind C1-inh. It was shown that aa 145-185 of CihC are responsible for this binding. Data suggests that C1-inh bound to CihC retains its functional activity and thus results in complement inactivation (Grosskinsky et al., 2010).

## Targeting complement at the CP and LP C3 convertase: C4BP recruitment

As previously mentioned, the spider-like C4BP acts as a co-factor for FI in the inactivation of C4b, thereby inhibiting formation of the CP and LP C3 convertase. In addition, it mediates dissociation of C2a from already formed C3 convertases (Rawal et al., 2009). Targeting of C4BP to a bacterial surface not only inhibits C3b deposition on the surface and subsequent phagocytosis. It eventually prevents lysis of Gram-negative bacteria by decreased formation of the MAC (Ermert and Blom, 2016). Bacterial recruitment of C4BP commonly involves interaction between the bacterial receptor and the C- or N-terminal SCRs of C4BP (Kraiczy and Würzner, 2006; Blom et al., 2009; Ermert and Blom, 2016). Bacteria share this latter binding site, SCR 1-2, with the host C4b protein therefore C4BP co-factor activity is unaffected (Ermert and Blom, 2016). *Haemophilus influenzae* is one of the pathogens known to bind C4BP. This Gram-negative respiratory pathogen is either classified as nontypeable (NTHi) or typable *influenzae* depending on the presence or absence of a polysaccharide capsule. *H. influenzae* type b (Hib) is the main serotype causing invasive disease leading to meningitis and osteomyelitis (Morris et al., 2008). The binding of C4BP to the bacterial surface of *H. influenzae* has been observed in most NTHi strains tested but not in the majority of typable strains (Hallström et al., 2007). Recruitment of C4BP on the bacterial surface was associated with decreased C3b deposition and increased serum resistance. The protein(s) responsible remain to be identified. Nonetheless, using C4BP mutants, the binding site on C4BP for NTHi binding, was narrowed down to the positively charged aa residues of SCR 2 and SCR 7 of the C4BP molecule (Hallström et al., 2007).

**Figure 4:**
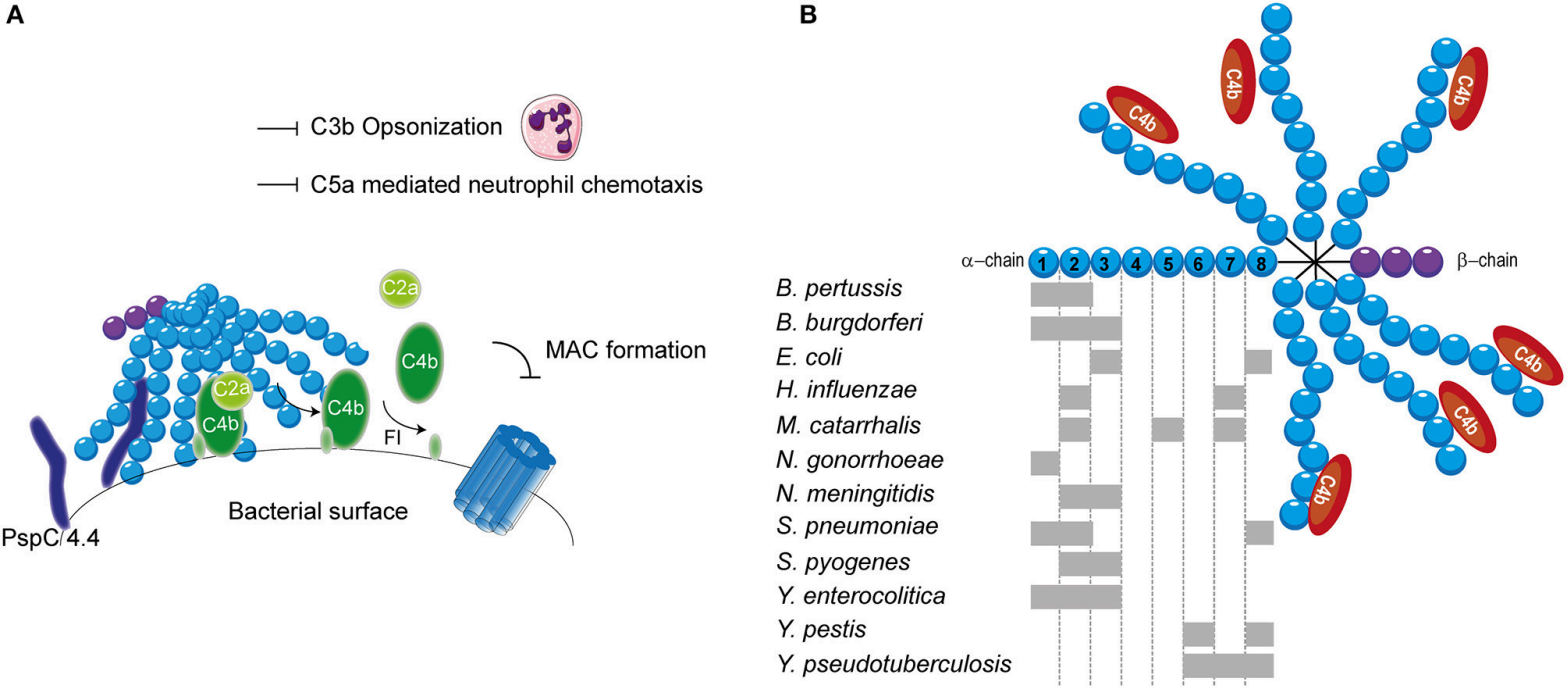
**C4BP recruitment by bacterial pathogens. (A)** PspC4.4 of *S. pneumoniae* recruits C4BP to the bacterial surface resulting in the decay of CP and LP C3 convertase and proteolytic cleavage of C4b. This subsequently inhibits formation of the MAC as well as decreases phagocytic uptake of the bacteria due to the inhibition of C3b deposition and C5a generation. **(B)** Multiple bacteria can bind the α-chain of C4BP at different sites as indicated by the gray rectangles. SCRs 1-3 of the α-chain mediate the interaction with C4b. Figure was produced using Servier Medical Art.

*Streptococcus pneumoniae*, has additionally been shown to bind to C4BP. *S. pneumoniae* is a Gram-positive commensal that colonizes the upper respiratory tract. Although often harmless, it can cause respiratory diseases such as otitis media, pneumonia and sinusitis (Cartwright, 2002). Furthermore, systemic diseases such as pneumococcal sepsis and meningitis can be caused by an infection with *S. pneumonia* (Mook-Kanamori et al., 2011). Surface exposed enolase, a pneumoccocal glycolytic enzyme, which can reassociate with the bacterial surface, was proposed as a C4BP binder (Agarwal et al., 2012). Due to the importance of this enzyme in glycolysis, mutant studies to verify the involvement of enolisin in evasion of complement mediated killing via the acquisition of C4BP cannot be performed. Nonetheless, enolase was shown to bind to SCRs 1-2 and SCR 8 and to limit C3b deposition (Agarwal et al., 2012). The autolytic enzyme LytA is the second C4BP recruiter of *S. pneumoniae*. Pneumococcal strains lacking the expression of LtyA bound less C4BP and were more sensitive to complement. Interestingly, LytA was shown to be important in the pathogenesis of sepsis and pneumonia in murine models (Ramos-Sevillano et al., 2015). In addition, clinical isolates have been shown to recruit varying levels of C4BP. Serotype 14 strains, which are associated with invasive human disease, showed the highest binding (Dieudonné-Vatran et al., 2009). This difference was attributed to the expression of a novel allelic variant of pneumococcal surface protein (Psp) C, namely PspC4.4, which was shown to bind C4BP as well (Dieudonné-Vatran et al., 2009). Taken together, *S. pneumoniae* recruits C4BP by interacting with the electropositive cluster at the SCRs 1-2 (Figure 4A). Additionally, SCR 8 is involved in the human-specific binding to C4BP (Dieudonné-Vatran et al., 2009; Agarwal et al., 2012).

The binding of C4BP to *B pertussis* is dependent on the expression of genes that are under the control of the Bordetella master virulence regulatory locus (BvgAS) (Berggård et al., 1997; Fernandez and Weiss, 1998). This two-component sensory transduction system regulates the expression of *bvg*-activated genes which include many known virulence factors such as pertactin, pertussis toxin, fimbriae, Vag8 and filamentous hemagglutinin (FHA) (Melvin et al., 2014). FHA has been proposed as the C4BP-binding protein of *B. pertussis* since FHA mutants bound less C4BP to their surface compared to wild type, however, C4BP binding was not abolished (Berggård et al., 1997). Nonetheless, FHA mutants displayed similar serum resistance as wild type strains suggesting that binding of C4BP via FHA does not mediate complement evasion (Fernandez and Weiss, 1998). FHA might facilitate the binding of a second protein to C4BP and thereby indirectly contribute to serum resistance (Berggård et al., 2001). Using C4BP mutants, SCRs 1-2, with a major role for the aa Arg64 and Arg66, was identified as the site responsible for binding to *B. pertussis* (Berggård et al., 2001).

Lyme disease is one of the major emerging arthropod borne infections in the world. This multi-systemic disease is caused by the Gram-negative *B. burgdorferi* senus lato complex including at least ten species. *B. burgdorferi* mainly causes disease in North America, whereas *Borrelia garinii* and *Borrelia afzelii* are the main causative agents of disease in Eurasia (Hengge et al., 2003). Infected *Ioxides* ticks, carrying the infectious agent in their mid-gut, infect humans during their blood meal (Hengge et al., 2003). Acute symptoms include a localized skin rash accompanied with headache, myalgia, arthralgia and fever. If left untreated, complications can become more severe and include chronic arthritis, commonly seen for *B. burgdorferi* infection and neurological abnormalities often associated with *B. garinii* (Hengge et al., 2003). As transmission from the arthropod vector to the host occurs via blood, *Borrelia* needs to effectively evade the complement system (Hengge et al., 2003). To this end, acquisition of C4BP to the bacterial surface is highly beneficial for survival. Indeed, C4BP recruitment has been reported for multiple pathogenic *Borrelia* species (Pietikäinen et al., 2010). Several potential mediators of C4BP acquisition of *B. burgdorferi* and *B. garinii* have been identified via mass spectrometry namely variable large protein, variable major protein and outer surface protein A (Pietikäinen et al., 2010; Madar et al., 2015). Both heparin and C4b addition disrupted the binding of C4BP to *B. burgdorferi* and *B. garinii*, indicating that the SCRs 1-3 with particular emphasis on SCR 2, which is most important for heparin binding, are important for this interaction (Pietikäinen et al., 2010). Therefore, it is likely that the positively charged aa at the interface between SCRs 1-2 are responsible for binding as is seen for most pathogens described here. In addition, *B. recurrentis* expresses CihC which is also able to bind C4BP. The central part of CihC, aa 145-185, was shown to bind to an unidentified part of C4BP via hydrophobic interactions (Grosskinsky et al., 2010).

Another Gram-negative bacterium which causes high level septicemia is *Yersinia pestis*, the causative agent of the plague. The attachment invasive locus (Ail) is important for adhesion and invasion of macrophages and dendritic cells by *Y. pestis* (Kolodziejek et al., 2012). Besides its involvement in cell adhesion, Ail was shown to mediate serum resistance of *Y. pestis* and to bind C4BP (Bartra et al., 2008; Ho et al., 2012). This high affinity interaction with a Kd of 0.76 nM involves SCRs 6 and 8 (Ho et al., 2014). The related *Yersinia enterocolitica*, causing enterocolitis, and *Yersinia pseudotuberculosis*, causing Far East scarlet-like fever also express Ail that can bind to C4BP (Kirjavainen et al., 2008; Ho et al., 2014). Like Ail of *Y. pestis, Y. pseudotuberculosis* Ail mediates C4BP recruitment via SCRs 6-8 (Ho et al., 2012). However, Ail of *Y. enterocolitica*, interacts with SCRs 1-3 of C4BP and is often masked by LPS making it incapable of contributing to C4BP recruitment (Kirjavainen et al., 2008). Hence, for *Y. enterocolitica*, a second surface protein namely YadA is largely responsible for C4BP binding. YadA interacts in a similar manner with C4BP than C4b where the binding site likely overlaps with SCRs 1-2 (Kirjavainen et al., 2008). The observation that different *Yersinia* species bind C4BP highlights the importance of C4BP recruitment for the survival of these pathogens (Ho et al., 2014).

The observation that C4b is rapidly cleaved on the *S. aureus* surface hinted toward the involvement of C4BP. Indeed, various strains, including multiple antibiotic resistant clinical isolates, displayed binding of C4BP to their surface (Hair et al., 2012). The staphylococcus surface protein SdrE of *S. aureus*, was shown to associate with C4BP (Hair et al., 2013). The *S. aureus* bone sialoprotein binding protein shares 75% sequence homology to SdrE and indeed also displays the capacity to bind C4BP (Hair et al., 2013). The regions involved in the binding between these two proteins and C4BP have not been identified. C4BP was shown to have a higher affinity for SdrE than *S. aureus* bone sialoprotein binding protein where the half-maximal binding for C4BP was seen at 15 nM/l and 30 nM/l respectively (Hair et al., 2013). An overview of the different bacterial species and their binding sites on C4BP is shown in Figure 4B.

## Inhibiting the AP: FH and FHL-1

As described in the introduction, the host protein FH is the main fluid phase regulator of the AP and is recruited by various pathogens as a complement evasion strategy. As previously mentioned, FH consists of 20 SCRs of which SCRs 1-4 are responsible for the regulatory activity of FH (Gordon et al., 1995). FH was the first discovered complement regulator to bind to bacteria (Horstmann et al., 1988). To evade the AP, bacteria commonly recruit FH to their surface by binding to SCRs 6-7 or SCRs 19-20 leaving the important active site of FH (SCRs 1-4) functional (Meri et al., 2013). Bacterial proteins that bind FH via SCRs 6-7 can also recruit FHL-1 due to conserved SCRs domains (Kühn et al., 1995). Bacterial proteins that utilize SCRs 19-20 bind to the so called “common microbial binding site” that consists of aa Arg1182, Arg1203 and Arg1206 of the SCR 20 (Meri et al., 2013). Both NTHi and typable *H. influenzae* strains are capable of recruiting FH to their surfaces (Hallström et al., 2008). Binding of FH was shown to vary between different strains and serum resistance correlates with the amount of FH recruited (Hallström et al., 2008). Typable *H. influenza* strains, especially Hib and *H. influenzae* type f (Hif), are more potent FH recruiters compared to the NTHi strains (Hallström et al., 2008). Moreover, four out of ten Hib strains tested also displayed adherence to FHL-1. Both SCRs 6-7 and SCRs 18-20 are shown to be involved in FH binding to Hib (Hallström et al., 2008). Protein H, is responsible for binding of FH to typable *H. influenza*, since deletion of Protein H resulted in decreased FH binding to the these bacteria (Fleury et al., 2014). The SCR 7 and SCRs 18-20 of FH are responsible for this interaction (Fleury et al., 2014). Binding of Protein H to FHL-1 was mediated via its SCR 7 (Fleury et al., 2014). NTHi strains do not express Protein H but can recruit FH via outer membrane protein P5 (OmpP5) (Rosadini et al., 2014; Langereis and de Jonge, 2015). OmpP5, a member of the OmpA protein family, is expressed in both NTHi strains as well as typable strains (Hill et al., 2001). Nonetheless, it is only actively involved in preventing serum killing via the AP in NTHis, most probably due to the sequence variations in OmpP5 (Hill et al., 2001; Rosadini et al., 2014; Langereis and de Jonge, 2015). Outer membrane loops 1 and 2 of OmpP5 contribute to the binding of FH via unidentified aa residues. Heterogeneity in these outer membrane loops have been reported and are associated with the levels of FH binding to specific strains (Langereis et al., 2014).

*S. pneumoniae* expresses several proteins that mediate attachment of FH to the bacterial surface and recruitment of this AP regulator correlates with the invasiveness of the strain (Hyams et al., 2013). Factor H binding inhibitor of complement (Hic), recruits FH via interactions between SCRs 8-11 and SCRs 12-14 of FH and aa 38-92 of Hic (Kohler et al., 2014). A second FH binding protein of *S. pneumoniae* is PspC, which specifically interacts with human FH SCRs 8-10 via aa 1-225 of its N-terminal hypervariable α helical domain (Dave et al., 2004; Herbert et al., 2015). Human specificity has been attributed to four different FH aa residues, namely Val495, Met497, Leu543 and Ile545, forming a so-called hydrophobic lock (Achila et al., 2015). PspC binds FH in a functionally enhanced conformation with a kD of 1 nM. This conformation results in a twofold enhanced binding of FH to C3b, which increases the decay of C3b with an impressive fivefold (Herbert et al., 2015).

The surface protein SdrE of *S. aureus*, previously described as a C4BP recruiter, was shown to additionally associate with FH and thus SdrE can inhibit all three complement pathways (Sharp et al., 2012). Upon binding of this protein, FH remains functionally active and is able to provide cofactor activity for FI to mediate subsequent cleavage of C3b to iC3b (Sharp et al., 2012). Furthermore, the previously mentioned homolog of SdrE, *S. aureus* bone sialoprotein binding protein, also displays the capacity to bind FH (Hair et al., 2013). The residues involved in the interaction between FH and SdrE or *S. aureus* bone sialoprotein binding protein have not been identified yet. Besides a membrane bound FH hijacker, there is evidence that *S. aureus* also expresses secreted proteins that enhance acquisition of FH to the surface (Amdahl et al., 2013). Ecb was shown to form a tripartite complex with C3b and FH via the SCRs 19-20 (as briefly described above) (Amdahl et al., 2013). The formation of such a complex had an enhancing effect on the function of both Ecb and FH, where Ecb facilitated the binding of FH to the bacterial surface. Furthermore, within this complex both proteins showed enhanced association to C3b (Amdahl et al., 2013). The tripartite complex successfully inhibited formation and shortened the half-life of the C3bBb convertase (Amdahl et al., 2013).

The most extensive studied protein with respect to FH binding is the human specific lipoprotein factor H binding protein (fHbp) of *Neisseria meningitidis*, which is a component of the recently licensed meningitidis type B vaccine (Seib et al., 2015). The human specific Gram-negative bacterium *N. meningitidis* is the main causative agent of invasive meningococcal disease and septicemia worldwide. Accordingly, it has evolved a number of serum resistance mechanisms (Schneider et al., 2007). The binding of FH and fHbp is a high affinity interaction with a kD of approximately 5 nM involving both the N- and C-terminal β barrel of fHbp and SCRs 6-7 of FH (Schneider et al., 2009). fHbp has been categorized into three different variant groups, V1, V2, and V3 based on sequence alignment (Masignani et al., 2003). Expression of fHbp is variable in clinical isolates (Biagini et al., 2016). Besides recruiting FH, fHbp is additionally capable of binding to FHL-1 and FHR3 (Caesar et al., 2014). FHR3 has been shown to be a competitive antagonist of FH (Józsi et al., 2015). Hence, recruitment of FHR-3 over FH would render a strain more easily killed by the AP. V1 strains have a lower affinity for FHR-3 compared to V3 strains (Caesar et al., 2014) as well as cause increased invasive disease (Lucidarme et al., 2009). A second FH binding protein of *N. meningitidis* is Neisserial surface protein A (NspA) which causes serum resistance even in the absence of fHbp (Lewis et al., 2010). NspA has an eight-stranded anti-parallel β-barrel structure with four putative surface exposed loops (Vandeputte-Rutten et al., 2003). Loop 3 appears to be important for binding to FH SCRs 6-7 (Lewis et al., 2010). Sialyation of lipooligosaccharides (LOS) is used as a strategy by many bacteria to mimic the human host and for *N. meningitidis* increases the binding of FH to NspA. The precise mechanism of this increased binding has not been resolved (Lewis et al., 2010). When investigated together, LOS sialic acid, fHbp and NspA were shown to cooperatively decrease C3 deposition through the AP on the bacterial surface (Lewis et al., 2012). Interestingly, *N. meningitidis* was still able to overcome complement mediated bacterial killing in the absence of fHbp, NspA and LOS. This remaining serum resistance and FH binding was attributed PorinB2 of *N. meningitidis* (Lewis et al., 2013). Furthermore, the meningococcal PorB3 has been implicated as an additional FH binding protein (Lewis et al., 2013).

**Figure 5:**
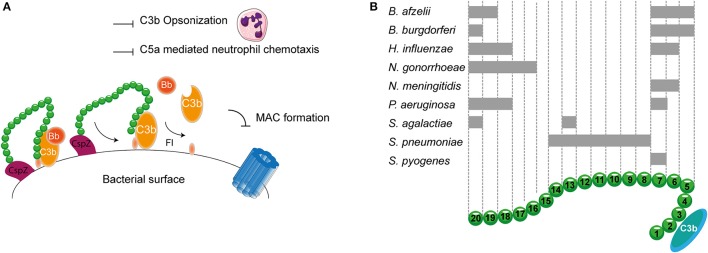
**FH binding by bacterial pathogens. (A)** One example of a FH binding bacterial protein is the surface protein CspZ of *B. burgdorferi*. CspZ interacts with SCR 20 of FH recruiting this AP regulator to the bacterial surface resulting in the decay of the AP C3 convertase and proteolytic degradation of C3b into iC3b and C3d. This subsequently inhibits formation of the MAC as well as decreases phagocytic uptake of the bacteria due to the inhibition of C3b deposition and C5a generation. **(B)** Multiple bacteria can bind FH at different sites as indicated by the gray rectangles. SCRs 1-4 mediate the interaction with C3b and SCRs 19-20 are involved in recognizing self from non-self. Figure was produced using Servier Medical Art.

As described before, transmission of *Borrelia* occurs from the arthropod vector to the host via blood. Therefore, *Borrelia* needs to effectively evade the complement system. To this end, it expresses multiple proteins that can bind to FH and FHL-1. CspA, CspZ, ErpA, ErpP, and OspE all mediate binding of *B. burgdorferi* to SCR 20 of FH (Figure 5A; Kenedy et al., 2009; Hallström et al., 2013). Furthermore, CspA CspZ and OspE are capable of interacting with the SCRs 5-7 of FHL-1. The crystal structures of ErpP, and OspE show a similar α and β domain structure made from eight anti-parallel β-strands and two short α-helices (Bhattacharjee et al., 2013; Caesar et al., 2013; Brangulis et al., 2015). Taken together, several bacterial species bind FH to their bacterial membrane. Their binding sites are summarized in Figure 5B.

## Inhibition of the terminal complement pathway: hijacking of Vn and clusterin

**Figure 6:**
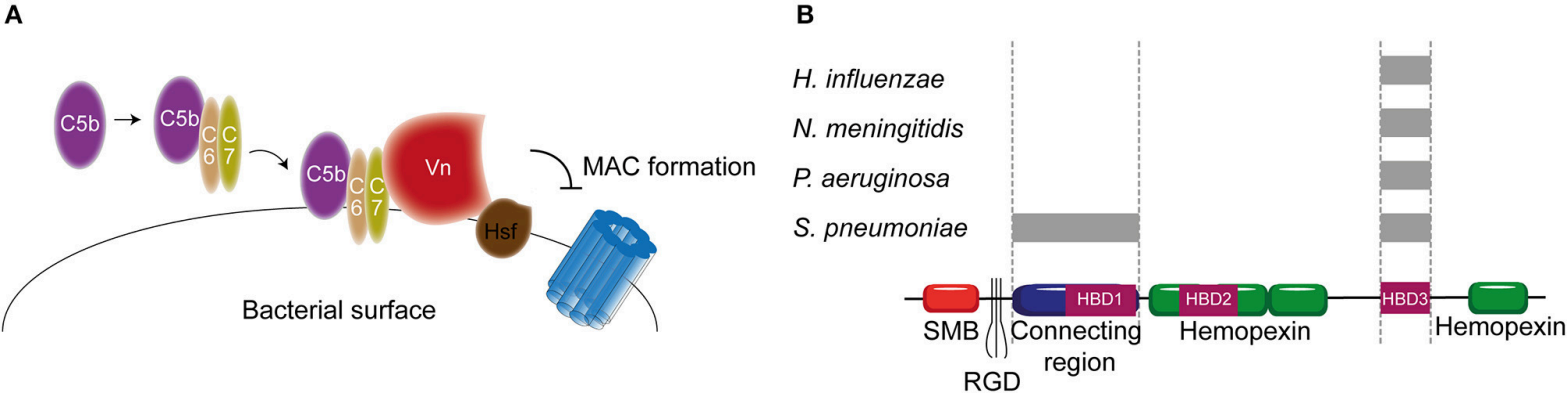
**Vn binding by bacterial pathogens. (A)** Hsf of *H. influenzae* binds to the HBD3 of Vn. By recruiting Vn to the bacterial surface, *H. influenzae* inhibits formation of the MAC. **(B)** Multiple bacteria can bind Vn at different sites as indicated by the gray rectangles. Bacterial pathogens either interact with Vn's connecting region or with the C-terminal HBD3. Figure was produced using Servier Medical Art.

As previously described, Vn prevents membrane insertion of the MAC by associating with the metastable membrane binding site of C5b-9 (Preissner, 1991). In a recent study, thirteen different microbial pathogens were analyzed for their Vn binding capabilities. Interestingly, all bacterial pathogens interacted with the same C-terminal heparin binding domain 3 (HBD3) (aa 352-374) of Vn indicating that binding of bacteria to Vn is highly conserved among various bacterial species (Hallström et al., 2016). *H. influenzae* is one of the pathogens known to bind Vn. Haemophilus Surface Fibrils (Hsf), which is highly conserved in typable strains, was the first identified Vn binding protein of *H. influenzae* (Hallström et al., 2006). Binding of Hsf to HBD3 of Vn was mediated via aa 429-652 of Hsf (Singh et al., 2014; Figure 6A). Protein H is another protein expressed by Hib, as well as by the newly emerging Hif, involved in mediating serum resistance through binding of Vn (Al-Jubair et al., 2015). NTHi strains have evolved to express distinct Vn-binding proteins namely Protein E and Protein F (Hallström et al., 2009; Singh et al., 2013; Su et al., 2013). Both proteins bind the HBD3 of Vn, but Protein E displays a stronger binding than Protein F. The centrally located aa 84-108 residues of Protein E interact with Vn (Hallström et al., 2009), while the N-terminal aa 23-48 residues of protein F make up for the Vn interaction site (Su et al., 2013). Besides *H. influenza, P. aeruginosa* is also known to express two proteins that are capable of binding Vn. The Vn binding proteins of *P. aeruginosa* Porin D and dihydrolipoamide dehydrogenase (Lpd) also bind to the HBD3 region of Vn (Hallström et al., 2015; Paulsson et al., 2015). In addition to Vn binding, Lpd can also interact with the second terminal complement pathway inhibitor clusterin (Hallström et al., 2015). Vn binds to aa 161-287 as well as aa 264-478 of Lpd, while clusterin binds to aa 1-160 and aa 328-478 of this protein (Hallström et al., 2015). Vn and clusterin where shown to bind simultaneously (Hallström et al., 2015). Interestingly, *P. aeruginosa* strains isolated from the airways show stronger binding capacity to Vn compared to strains isolated from the blood stream (Paulsson et al., 2015). Whether the amount of Porin D or Lpd expressed by these strains can account for the different binding strengths and whether these strains are more serum resistant remains elusive.

PspC and Hic, previously discussed as FH binding protein, have additionally been identified as the *S. pneumoniae* Vn binding molecules with MAC inhibiting properties (Voss et al., 2013; Kohler et al., 2014). Vn binding to PspC is mediated via the N-terminal region of PspC, which binds to the HBD3 of activated Vn (Voss et al., 2013). Hic, also known as PspC11.4, binds to activated Vn with its central part (aa 151-200), suggesting it has adopted a distinct binding mechanism compared to the classical PspC protein. Nonetheless, the binding regions on activated Vn is similar to PspC (Kohler et al., 2014). Outer membrane protein Opc and Meningococcal Surface Fibril (Msf) have been identified as the Vn binding proteins of *N. meningitidis* that are variably expressed among isolates and associated with increasing serum resistance (Griffiths et al., 2011). Opc is a transmembrane protein of the β barrel family with five surface exposed loops. Initially, the binding of Opc to Vn was solely described in the context of enhanced adhesion to human brain endothelial cells (Sa E Cunha et al., 2010). Subsequently, studies linked Opc expression to serum resistance (Hubert et al., 2012). Opc can bind to two distinct sites on Vn namely the connecting region and the HBD3 region. This binding is mediated by sulphated tyrosines on Vn or bridged by heparin respectively (Sa E Cunha et al., 2010). The residues on Opc responsible for Vn binding have not been identified yet. Like Opc, Msf binds similar sites on the activated Vn however, unlike Opc, this binding does not depend on sulphated tyrosines in the Vn molecule nor heparin (Griffiths et al., 2011). Molecular modeling has identified aa 39-82 of Msf to be important for Vn binding (Hill et al., 2015). Figure 6B summarizes the different pathogens and their binding places to Vn. In addition, Table 2 shows an overview of all the complement evasion molecules, that specifically bind complement regulatory proteins, described in this review in combination with the previously reviewed molecules (Kraiczy and Würzner, 2006; Blom et al., 2009).

## Complement evasion molecules as vaccine targets

Numerous successful vaccines are in use today, however for many bacteria and diseases a vaccine has not been developed yet (Rappuoli and Medaglini, 2014). The lack of available vaccines against several bacterial species such as *S. aureus*, and *B. burgdorferi* is of great concern due to the emergence of multidrug resistant strains and high Lyme disease prevalence, respectively (Missiakas and Schneewind, 2016; Plotkin, 2016). Since traditional vaccine development is failing for these bacteria, it is of high importance to find (new) protein candidates. A bacterial protein is considered a good vaccine candidate if it is immunogenic, highly conserved within the targeted bacterial species and has suitable physicochemical properties (Meri et al., 2008). Complement evasion molecules fulfill most, if not all, of these criteria. Studies show immunogenicity of multiple complement evasion molecules in vaccination models including *B. pertussis* Vag8, *S. pneumoniae* PspC and *S. aureus* SdrE (Stranger-Jones et al., 2006; Ferreira et al., 2009; de Gouw et al., 2014). Furthermore, the vast majority of the complement evasion molecules described in this review are expressed by most, if not all, virulent strains of one particular bacterial species and the proteins are highly conserved (Bhattacharjee et al., 2013; Fleury et al., 2014; de Gouw et al., 2014; Hallström et al., 2015).

An additional reason to take complement evasion molecules into consideration as good vaccine candidates is the fact that antibodies raised against these protein might contain blocking activity (Serruto et al., 2010). Therefore, recruitment of C1-inh, C4BP, FH, or Vn to the bacterial surface might be inhibited and subsequently increase bacterial susceptibility to complement-mediated killing. Moreover, increased complement activation would result in the generation of the chemoattractant C5a resulting in increased phagocytosis and increased bacterial killing (Serruto et al., 2010). However, other reports have shown that the binding of host molecules to vaccine targets can result in the induction of the antibodies directed against parts of the vaccine target that are not involved in the binding to the complement regulatory protein. This phenomenon can be overcome by the use of mutated bacterial proteins that are no longer able to bind their host target protein (Rossi et al., 2013; Zariri et al., 2013; Costa et al., 2014; Granoff et al., 2016). Therefore, the use of mutated complement evasion molecules that no longer bind to their host target proteins should be considered in the design of microbial vaccines that aim to elicit antibodies that block binding of the host complement regulator to the pathogen. Another important aspect of complement evasion molecules is that a large proportion of these molecules bind to their ligands in a strictly human-specific manner (Pan et al., 2014). Traditionally, the immunogenicity of potent vaccine targets is tested in mice. These antibodies are then assessed for their ability to, for instance, interfere with the binding of complement molecules to the vaccine target (De Gregorio and Rappuoli, 2014). Due to the human specificity of a lot of the complement evasion molecules this needs to be considered for vaccine development since the data obtained in mice might not correspond with data obtained in humans.

Besides protein based vaccines, outer membrane vesicles (OMVs) may serve as a vaccine platform as the vast majority of complement regulatory protein binding bacterial proteins are displayed on the surface. Recent advances in bacterial engineering allow for the customization of OMVs (van der Pol et al., 2015). Strategies include the introduction of heterologous proteins in OMVs as well as retaining antigens which normally undergo proteolytic processing and are released from the bacterial membrane (Kuipers et al., 2015; van der Pol et al., 2015). The effectiveness of complement evasion molecules in vaccines is proven by *N. meningitidis* fHbp and *B. pertussis* FHA (Santolaya et al., 2012; Locht, 2016). Vaccination with fHbp results in the production of antibodies that prevent FH binding to the bacterial surface (Serruto et al., 2010). In this review, several bacterial proteins have been discussed that have the ability to recruit multiple host complement regulatory proteins. The best example of this is the Lpd protein of *P. aeruginosa*. This protein is a master in binding complement regulatory proteins recruiting Vn, Clusterin and FH simultaneously (Hallström et al., 2015). Furthermore, PspC of *S. pneumoniae* can bind Vn and FH. Additional proteins that bind multiple regulatory proteins include *H. influenzae* Protein H, *S. aureus* SdrE, *S. pneumoniae* Hic and *Yersinia* Ail (Sharp et al., 2012; Hair et al., 2013; Fleury et al., 2014; Ho et al., 2014; Kohler et al., 2014; Al-Jubair et al., 2015). Proteins that bind multiple complement regulators to the bacterial surface will be of high importance to bacterial survival. Therefore, these proteins might be very effective as vaccine candidates as antibodies raised against these proteins will likely mediate the loss of the ability to bind not one, but multiple complement regulatory proteins to the surface of the targeted bacteria.

As discussed above, the use of bacterial proteins as vaccine antigens is a strategy that has successfully been implemented for multiple vaccines including the two vaccines mentioned above as well as the *H. influenzae* and pneumococcal vaccines. One potential pitfall of this vaccine strategy is that bacteria can evolve to circumvent vaccine induced immunity. For example, a high percentage of the currently circulating *B. pertussis* strains no longer express the vaccine antigen pertactin (Hegerle and Guiso, 2014; Locht, 2016). Another example is the capsular switching observed for *S. pneumoniae* (Brueggemann et al., 2007). To this end, vaccine improvement is needed and combining several complement evasion molecules that in turn can bind multiple host complement regulatory proteins could minimize potential bacterial vaccine-escape and yield an interesting candidate vaccine. Concluding, elucidating the interactions between bacterial proteins and host complement regulatory proteins provides valuable information needed for the development of new vaccines for non-vaccine preventable diseases as well as to improve currently used vaccines.

## Author contributions

EH, Bv, and IJ wrote the manuscript, drafted the figures and approved its final version.

### Conflict of interest statement

The authors declare that the research was conducted in the absence of any commercial or financial relationships that could be construed as a potential conflict of interest.
